# Operational Performance of a Plasmodium falciparum Ultrasensitive Rapid Diagnostic Test for Detection of Asymptomatic Infections in Eastern Myanmar

**DOI:** 10.1128/JCM.00565-18

**Published:** 2018-07-26

**Authors:** Jordi Landier, Warat Haohankhunnatham, Smita Das, Kamonchanok Konghahong, Peter Christensen, Jathee Raksuansak, Pase Phattharakokoedbun, Ladda Kajeechiwa, May Myo Thwin, Ihn Kyung Jang, Mallika Imwong, Jacher Wiladphaingern, Khin Maung Lwin, Clare Ling, Stephane Proux, Gonzalo J. Domingo, Gilles Delmas, François H. Nosten

**Affiliations:** a Shoklo Malaria Research Unit, Mahidol-Oxford Tropical Medicine Research Unit, Faculty of Tropical Medicine, Mahidol University, Mae Sot, Thailand; b Institut de Recherches pour le Développement (IRD), Aix Marseille Université, INSERM, SESSTIM, Marseille, France; c PATH, Diagnostics Program, Seattle, Washington, USA; d Mahidol-Oxford Tropical Medicine Research Unit, Faculty of Tropical Medicine, Mahidol University, Bangkok, Thailand; e Centre for Tropical Medicine and Global Health, Nuffield Department of Medicine, University of Oxford, Oxford, United Kingdom; Carter BloodCare & Baylor University Medical Center

**Keywords:** Plasmodium*falciparum*, asymptomatic infection, low density parasitemia, low transmission setting, malaria, rapid tests, ultrasensitive RDT

## Abstract

In the Greater Mekong Subregion in Southeast Asia, malaria elimination strategies need to target all Plasmodium falciparum parasites, including those carried asymptomatically. More than 70% of asymptomatic carriers are not detected by current rapid diagnostic tests (RDTs) or microscopy. An HRP2-based ultrasensitive RDT (uRDT) developed to improve the detection of low-density infections was evaluated during prevalence surveys within a malaria elimination program in a low-transmission area of eastern Myanmar. Surveys were conducted to identify high-prevalence villages. Two-milliliter venous blood samples were collected from asymptomatic adult volunteers and transported to the laboratory. Plasmodium parasites were detected by RDT, uRDT, microscopy, ultrasensitive qPCR (uPCR), and multiplex enzyme-linked immunosorbent assay (ELISA). The sensitivity, specificity, and predictive positive and negative values of RDT and uRDT were calculated compared to uPCR and ELISA. Parasite and antigen concentrations detected by each test were defined using uPCR and ELISA, respectively. A total of 1,509 samples, including 208 P. falciparum-positive samples were analyzed with all tests. The sensitivity of the uRDT was twofold higher than that of RDT, 51.4% versus 25.2%, with minor specificity loss, 99.5% versus 99.9%, against the combined reference (uPCR plus ELISA). The geometric mean parasitemia detected by uRDT in P. falciparum monospecific infections was 3,019 parasites per ml (95% confidence interval [95% CI], 1,790 to 5,094; *n* = 79) compared to 11,352 parasites per ml (95% CI, 5,643 to 22,837; *n* = 38) by RDT. The sensitivities of uRDT and RDT dropped to 34.6% and 15.1%, respectively, for the matched tests performed in the field. The uRDT performed consistently better than RDT and microscopy at low parasitemias. It shows promising characteristics for the identification of high-prevalence communities and warrants further evaluation in mass screening and treatment interventions.

## Abstract (precis)

In the Greater Mekong Subregion in Southeast Asia, malaria elimination strategies need to target all Plasmodium falciparum parasites, including those carried asymptomatically. More than 70% of asymptomatic carriers are not detected by current rapid diagnostic tests (RDTs) or microscopy.

## INTRODUCTION

Elimination of Plasmodium falciparum malaria is a major goal for all countries in Southeast Asia, and specifically in the Greater Mekong Subregion (GMS), where antimalarial resistance threatens the progress made over the last 15 years (1). An increasing number of countries outside Southeast Asia are also committing to malaria elimination by 2030 (2).

In the GMS, reports of large numbers of asymptomatic malaria infections not detected by standard rapid diagnostic test (RDT) or microscopy (3) and insufficient protection provided by long-lasting insecticide-treated nets (LLINs) (4, 5) led to the development of P. falciparum elimination interventions which mainly focus on the prevention of human transmission (6–8): prompt treatment of clinical episodes with effective artemisinin combination therapy (ACT) and a single low dose of primaquine as a gametocytocidal agent (9) and identification and treatment of asymptomatic carriers (7). In low-transmission settings, these individuals can successfully transmit parasites to mosquitos even when they are infected at low-density parasitemias (10, 11). Recent evidence from the GMS also suggests that 20% of asymptomatically infected individuals may carry their infection for ≥4 months (12).

When asymptomatic carriers are tested by point-of-care methods such as microscopy and RDT in low-transmission settings, the infection is often not detected because these individuals generally harbor low parasite densities and P. falciparum antigen concentrations, respectively (3, 13, 14). In the GMS, a series of prevalence surveys were conducted using high-volume ultrasensitive quantitative PCR (uPCR) detection. According to the observed distribution of P. falciparum parasitemias, more than 80% of all P. falciparum-infected carriers harbor infections with parasite densities less than 100,000 parasites/milliliter (p/ml) (3). Consequently, surveys relying on standard RDT, which reliably detects parasitemias above 100,000 p/ml identify only 20% of the distribution and are unable to estimate community prevalence with sufficient precision (3, 15). Likewise, screening and treatment or focal mass drug administration (MDA) interventions relying on RDT conducted in low-incidence settings have missed significant numbers of cases and yielded disappointing results (14, 16, 17). Highly sensitive laboratory methods such as uPCR require central laboratories and skilled staff and are costly (3, 18). The delay between sample collection and results excludes their use for screening and treatment interventions. Even less constrained molecular methods with intermediate sensitivity (i.e., loop amplification-mediated PCR [LAMP]) often remain too complex or demanding to be deployed routinely in the remote areas where malaria persists (19).

The identification of low-density parasite carriers is therefore an important bottleneck to current interventions. The need for tools to bridge the gap in sensitivity between point-of-care tests and laboratory methods resulted in a new generation of tests, including the Alere Malaria Ag P.f Ultra Sensitive HRP2-based ultrasensitive RDT (uRDT) (20, 21). It can be anticipated that this is the first of a new generation of more sensitive malaria RDTs that will become available to malaria programs. Previously the uRDT was evaluated only under laboratory conditions with frozen or cultured samples (20, 21).

The objective of this study was to confirm the sensitivity, specificity, and detection properties of the uRDT for the detection of low-density P. falciparum infections in asymptomatic individuals in eastern Myanmar. This region of low and seasonal malaria incidence is currently targeted for malaria elimination and could benefit from rapid deployment of the uRDT if the performance characteristics are adequate (22).

## MATERIALS AND METHODS

### Context.

From 11 June 2016 to 17 January 2017, the evaluation of the uRDT was included prospectively in routine baseline surveys conducted in 39 villages in Hpapun Township, Kayin State, Myanmar, to identify high-prevalence villages as part of the Malaria Elimination Task Force (METF) program (22). The surveys included two steps: (i) an exhaustive RDT and uRDT screening of the village population and (ii) collection of venous blood samples from a random subsample of adult village inhabitants. The data presented here correspond to field and laboratory results obtained for the subsample participating in the two steps.

### Participants.

Participants were the inhabitants of the villages selected randomly or targeted for a prevalence survey within the METF program (22). Survey teams took samples from adults who agreed to participate, attempting to balance samples across sex and broad age groups, until reaching the sample size needed based on the full village population (see below). This sample size represented a significant proportion of the village population. Assuming that 50% of inhabitants were older than 18, which was verified in a complete census obtained during MDA interventions, the sample size often comprised between 30 and 50% of adult village population. Blood samples (2-ml samples of venous blood) were taken from adults who provided informed consent for laboratory analyses. Individuals with an axillary temperature of ≥37.5°C or reporting a history of fever in the previous 2 days were excluded from the analysis.

### Ethics statement.

First, community engagement teams sought community approval ahead of the survey date. Then, survey participants received individual information in their language, and informed consent was obtained from each individual before they provided a venous blood sample. Appropriate treatment for Plasmodium falciparum or Plasmodium vivax was available for all RDT-positive individuals.

The METF project has ethical approval from the Lower Myanmar Department of Medical Research Ethics' committee (reference 73/ETHICS2014, dated 25 November 2014, and renewed in November 2015 and 2016 under the same reference). The specific use of a 40-μl whole-blood aliquot for supplementary control for HRP2 detection was approved by the Oxford Tropical Research Ethics Committee (OxTREC 516-17). A favorable opinion was also expressed by representatives of the communities in the Thailand-Myanmar border region within the Tak Community Advisory Board (TCAB-09/REV/2016).

### Blood collection and handling.

A 2-ml venous blood sample was collected from each participant in an EDTA Vacutainer. After homogenization, venous samples were stored at 4°C in a cooler box and transported to the Shoklo Malaria Research Unit (SMRU) laboratory in Mae Sot, Thailand, within 24 h from sample collection.

In the laboratory, 5 μl whole blood was used to perform one normal RDT, 5 μl whole blood was used to perform one uRDT, and approximately 10 μl was used to perform a thick blood smear of standardized thickness (6 μl spread on a 12-mm circle) and a thin blood smear. A 40-μl aliquot was kept at −80°C for HRP2 detection using the Quansys ELISA reference assay. The remaining whole blood was centrifuged, 500 μl packed red blood cells (RBC) were used for the ultrasensitive qPCR detection, and the remaining aliquot of packed RBC was stored at −80°C.

### RDT and uRDT testing.

All whole-blood specimens were tested in singlet according to the manufacturer's instructions, using the SD BIOLINE Malaria Ag P.f. P.v. RDT (Standard Diagnostics, Inc./Alere, Republic of Korea) referred to hereafter as “RDT” and using the Alere Malaria Ag P.f. Ultra Sensitive uRDT (manufactured by Standard Diagnostics, Inc., Republic of Korea) referred to hereafter as “uRDT.”

In the field, survey team members performed the uRDT and RDT at the sample collection site for each participant providing a venous blood sample. The results were interpreted by a single reader in a nonblind manner as invalid (no control line or no proper clearance of the test), positive (control line and test line present), or negative (control line present, no test line) without reporting signal intensity.

In the laboratory, experienced laboratory technicians blind to field test results performed the RDT and uRDT. The uRDT was performed before the RDT and read by two independent readers who were blind to each other's reading results and to the results of all other tests conducted in the laboratory. The results were interpreted as invalid (no control line or no proper clearance of the test), positive (control line and test line present), or negative (control line present, no test line). Furthermore, each laboratory reader graded the strength of the signal in tests interpreted as positive in four categories: inconclusive (or “doubtful”, intensity of 1 or +/−−), faint positive (intensity of 2 or +/−), positive (intensity of 3 or +), and strong positive (intensity of 4 or ++). Intensities 2 to 4 corresponded to unambiguous results and were defined as “conclusive” in the analysis. A third reader interpreted the test result if the two prior readers disagreed on the test result.

### Microscopy.

Thick smears were declared negative if no parasites were seen in 200 high-power fields (HPF) (total magnification, ×1,000), equivalent to 0.27 μl of blood examined.

### Reference assays.

Three references were used to estimate the performance of the uRDT, RDT, and microscopy (Table 1).

**TABLE 1:** Definitions of reference methods

uPCR result^*a*^	Quansys ELISA result^*b*^							
	PfHRP2 detected				PfHRP2 not detected			
	P. falciparum DNA	P. vivax DNA	Plasmodium DNA	Plasmodium DNA not detected (negative)	P. falciparum DNA	P. vivax DNA	Plasmodium DNA	Plasmodium DNA not detected (negative)
Reference A	+	+	+	+	−	−	−	−
Reference B	+	−	−	−	+	−	−	−
Reference C	+	+	+	−	+	−	−	−

a Reference A corresponds to Quansys ELISA result only (positive if and only if P. falciparum HRP2 [PfHRP2] antigen present). Reference B corresponds to uPCR result only (positive if and only if P. falciparum DNA present). Reference C combines uPCR and Quansys ELISA (positive if P. falciparum DNA present or if PfHRP2 and Plasmodium DNA [Plasmodium spp. or P. vivax] present).

b A sample is considered positive (+) (or negative [−]) according to the reference.

### Quansys human malaria 4-plex ELISA (reference A).

Reference antigen detection and quantification by Quansys ELISA (Quansys Biosciences, Logan, UT) were conducted at the PATH laboratory (Seattle, WA) using the 40-μl whole-blood aliquot stored at −80°C following methods described previously (20). Three Plasmodium antigens in a sample were detected by the Quansys human malaria 4-plex ELISA: P. falciparum histidine-rich protein 2 (PfHRP2), P. vivax lactate dehydrogenase (PvLDH), and Plasmodium LDH (PanLDH). All assays are performed in the same well for each clinical specimen (unpublished results). Samples were defined as PfHRP2 antigen positive if the PfHRP2 concentration was measured above 2.3 pg/ml by Quansys ELISA.

### High-volume ultrasensitive qPCR approach (reference B).

The method for high-volume ultrasensitive qPCR (uPCR) detection of malaria parasites with a lower limit of detection of 0.02 parasites/μl was performed as described previously (18, 22). Briefly, the detection was conducted in two steps. First, a high-volume ultrasensitive qPCR detecting and quantifying Plasmodium genus parasites was performed in duplicate to quantify the parasite concentration. Samples exhibiting a discrepant result or a difference of >2 cycle threshold (*C_T_*) between the two duplicates were reanalyzed. Samples exhibiting a second discrepant result between duplicates upon reanalysis were interpreted as undetermined and excluded from the analysis. Second, two species identification single-target real-time PCR (RT-PCR) targeting species-specific sequences of P. falciparum and P. vivax were performed on positive samples.

### Combined reference (reference C).

The combined results of Quansys ELISA and uPCR were used at the third reference assay to evaluate the performance of the uRDT. This combined reference improved the species determination of each assay. Samples were defined as positive for P. falciparum according to reference C if P. falciparum-specific DNA was detected by uPCR or if Plasmodium or P. vivax DNA was detected by uPCR together with PfHRP2 by Quansys ELISA.

### Data management.

Demographic data (age, sex, and fever status) on surveyed participants were recorded in the field on paper forms. Each participant providing a venous blood sample was associated with a preprinted label sticker that included a unique identifier (consisting of a sample order number and a village code) printed clearly and in a barcode. A preprinted sticker was added to each participant's demographic data form and sample tube. All laboratory tests (uRDT, RDT, microscopy, and uPCR) were conducted using the unique identifier or barcode. The paper data were entered twice into a Microsoft Access database, and all discrepant values were checked against the original forms.

### Statistical analyses.

Statistical analysis was conducted using STATA 14.1 (Stata Corp., College Station, TX, USA). For each reference method, the sensitivity, specificity, positive predictive value, and negative predictive value were calculated. Binomial 95% confidence intervals (95% CI) were calculated. Quantitative variables (parasitemia or PfHRP2 concentration) were described by their mean and interquartile range (IQR) and were transformed by logarithm base 10 for graphic representation. All parasite densities were expressed in parasites per milliliter (p/ml).

The proportion of positive tests per category of parasite density (measured by uPCR) was calculated for samples with a single P. falciparum infection. The continuous probability of obtaining a positive test according to P. falciparum parasitemia was estimated by logistic regression, using the decimal logarithm of parasitemia (in parasites per milliliter) to predict the test result for each test (uRDT, RDT, field uRDT, or field RDT). Samples with Plasmodium DNA and PfHRP2 (positive by reference C but not reference B) or with mixed P. falciparum and P. vivax DNA detected by uPCR were excluded from these analyses: the quantification of parasitemia conducted during the genus-specific step of the uPCR did not distinguish P. falciparum-specific parasitemia from P. vivax or other potentially coinfecting species.

### Sample size.

The sample size for the small-sample venous blood survey was calculated in order to identify a malaria prevalence of 40% by uPCR with a 90% confidence interval of 10%. The number of samples required was between 30 and 65 samples per village (22).

The number of samples required to evaluate the performance of uRDT compared to uPCR was calculated based on its specifications (detection of parasitemia above 10 parasites/μl) and previous evaluation (20). The uRDT was expected to identify about 50% of uPCR-positive samples. To estimate a 50% sensitivity compared to uPCR, with a 95% confidence interval of 5%, the sample size required was 250 P. falciparum uPCR-positive samples, which required collecting 2,500 samples in Hpapun township (P. falciparum uPCR prevalence of ∼10% across 143 surveys [23]).

## RESULTS

### Overview.

**FIG 1:**
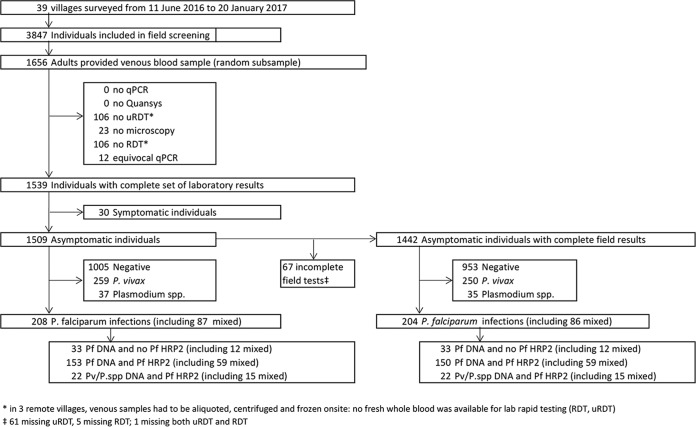
Flowchart of study participants and results. Species results obtained by the combination of Plasmodium DNA detection by uPCR and Plasmodium antigens by Quansys ELISA (reference C) are displayed.

Across 39 villages surveyed, 1,656/3,847 (43%) adult participants were randomly selected to provide venous blood. Of these 1,656 samples, 1,509 samples from asymptomatic participants from 36 villages had a result with each of the five laboratory malaria detection tests and were included in the analysis (Fig. 1). The median age of participants was 36 years (IQR, 27 to 48 years; *n* = 1,507), and the proportion of female participants was 52.9% (798/1,509). A median of 45 samples per village (IQR, 33 to 50 samples) was collected.

Combined results of uPCR analysis and Quansys ELISA (reference C) identified 1,005 negative samples, 259 P. vivax infections, 121 P. falciparum infections, 87 mixed P. falciparum and P. vivax infections, and 37 Plasmodium infections of undetermined species (Fig. 1).

### uRDT detection of asymptomatic P. falciparum infections in the laboratory.

For the detection of P. falciparum infections (reference C), the sensitivity of the uRDT was 51.4% (95% CI, 44.4 to 58.4%) with a specificity of 99.5% (95% CI, 98.9 to 99.8%) (Table 2). The sensitivity and specificity of the uRDT were similar compared to the other references (Table 2): 52.0% (95% CI, 44.9 to 59.0%) and 99.3% (95% CI, 98.7 to 99.7%) for PfHRP2 detection (reference A), respectively, and 53.8% (95% CI, 46.3 to 61.1%) and 98.9% (95% CI, 98.2 to 99.4%) for P. falciparum DNA-positive samples (reference B), respectively. In comparison, the sensitivities of RDT and microscopy were similar and low (ranging from 23.6 to 27.4% [Table 2]). As a result, uRDT consistently exhibited a twofold increase in sensitivity compared to the sensitivity of RDT and microscopy.

**TABLE 2:**
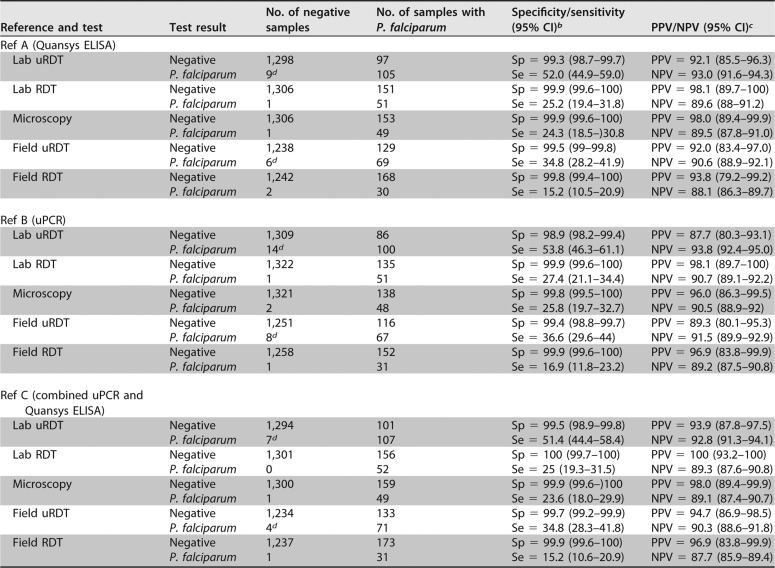
Specificity, sensitivity, and positive and negative predictive values for uRDT, standard RDT, and microscopy compared to reference methods^*a*^

a The references (Ref) are combined reference uPCR plus Quansys (PfDNA or Plasmodium DNA plus PfHRP2), reference P. falciparum DNA detection by uPCR, and reference PfHRP2 detection by Quansys.

^*b*^The specificity (Sp) and sensitivity (Se) and 95% confidence interval are shown.

^*c*^The positive predictive value (PPV) and negative predictive value (NPV) and 95% confidence interval are shown.

^*d*^See Table 3 for details on uRDT false-positive samples.

In total, 18 samples were positive for P. falciparum by uRDT in the laboratory and negative by at least one reference (references A to C) (Table 3). Only 5/18 (28%) uRDT-positive samples were negative by all references and corresponded to “true” false-positive results: 3 samples contained neither detected Plasmodium DNA nor antigens, 1 sample was P. vivax positive by uPCR and malaria negative by Quansys ELISA, and 1 sample was P. vivax positive by uPCR and Quansys ELISA. Among the remaining samples, 4/18 (22%) were positive by reference B and C, 7/18 (39%) were positive by reference C only, and 2/18 (11%) were positive by reference A only.

**TABLE 3:** Number of samples per reference result for all samples with a P. falciparum-positive uRDT and a P. falciparum-negative result for at least one reference (of references A to C)^*a*^

Test location and Quansys ELISA result (type of antigens identified)^*b*^	No. of samples with uPCR result (type of DNA identified)^*b*^			
	None	P. vivax	Plasmodium spp.	P. falciparum
Laboratory				
None	3^*c*^	1^*c*^	0^*c*^	2^*f*^
P. vivax (PvLDH)	0^*c*^	1^*c*^	0^*c*^	0^*f*^
P. falciparum (PfHRP2)	2^*d*^	0^*e*^	4^*e*^	NA
P. falciparum and *P. vivax (*PfHRP2 plus PvLDH)	0^*d*^	1^*e*^	2^*e*^	NA
Plasmodium spp. (PanLDH only)	0^*c*^	0^*c*^	0^*c*^	2^*f*^
Field				
None	1^*c*^	1^*c*^	0^*c*^	0^*f*^
P. vivax (PvLDH)	0^*c*^	2^*c*^	0^*c*^	1^*f*^
P. falciparum (PfHRP2)	0^*d*^	1^*e*^	3^*e*^	NA
P. falciparum and *P. vivax (*PfHRP2 plus PvLDH)	0^*d*^	0^*e*^	0^*e*^	NA
Plasmodium spp. (PanLDH only)	0^*c*^	0^*c*^	0^*c*^	1^*f*^

a A color-coded version of Table 3 is available in the supplemental material (Table S1).

b See footnotes *c* to *f* below. Values with footnotes *c* and *d* are false-positive results by reference C (*n* = 7 for laboratory and *n* = 4 for field). Values with footnotes *c* and *f* are false-positive results by reference A (*n* = 9 for laboratory and *n* = 6 for field). Values with footnotes *c*, *d*, and *e* are false-positive results by reference B (*n* = 14 for laboratory and *n* = 8 for field). NA, not applicable (corresponds to PfHRP2-positive and PfDNA-positive samples [true positive according to all references]).

c P. falciparum-negative samples by all three reference methods. These are most likely false-positive uRDT results.

d PfHRP2-positive, PfDNA-negative, and Plasmodium DNA-negative samples (positive by reference A only). These two samples could correspond to recently cleared infections with persisting HRP2 antigenemia.

e PfHRP2-positive, PfDNA-negative, and Plasmodium DNA-positive samples (P. falciparum positive by reference A and C).

f PfHRP2 negative and PfDNA positive (P. falciparum positive by reference B and C).

### Direct comparison of uRDT with standard RDT and microscopy in the laboratory.

uRDT detected 100% (52/52) of RDT-positive P. falciparum infections (reference C), while RDT detected only 49% (52/107) of uRDT-positive infections. Likewise, uRDT detected 98% (48/49) of microscopy-positive infections, while microscopy detected only 45% (48/107) of uRDT-positive infections.

### uRDT detection of asymptomatic P. falciparum infections in the field.

Overall, 1,442 asymptomatic adults with results for all five laboratory tests had also been tested in the field by uRDT and RDT (Fig. 1). The sensitivity of field testing was lower than in the laboratory for both uRDT and RDT, with respective sensitivities of 34.8% (95% CI, 28.3 to 41.8%) and 15.2% (95% CI, 10.6 to 20.9%) compared to reference C (Table 2). The uRDT in the field remained twofold more sensitive than the RDT compared to all three references (Table 2). The specificity of the tests conducted in the field was not significantly different from the tests conducted in the laboratory, 99.7% (95% CI, 99.2 to 99.9%) for uRDT and 99.9% (95% CI, 99.6 to 100%) for RDT.

In the field, there were 10 positive uRDT results corresponding to at least one negative reference test (Table 3). Four samples were negative by all reference methods, while two samples were positive according to reference B and C and four samples were positive according to reference C.

### Direct comparison of field uRDT with laboratory uRDT.

Out of 1,442 samples with complete field and laboratory results, 75 were positive by uRDT in the field, and 113 were positive by uRDT in the laboratory. Field and laboratory results agreed in 67 positive samples and 1,321 negative samples. Eight samples were positive only by field uRDT, of which four were also positive by at least one reference method and four were negative by all reference methods. Conversely, 46 samples were positive in the laboratory but not in the field, of which 41 were also positive by at least one reference method and five were negative by all references.

The sensitivity of field testing compared to the same test in the laboratory was 59.2% (95% CI, 49.6 to 68.4%) for uRDT and 60.8% (95% CI, 46.1 to 74.2%) for RDT. The corresponding specificity was 99.4% (95% CI, 98.8 to 99.7%) for uRDT and 99.9% (95% CI, 99.6 to 100%) for RDT.

### Quantitative detection of parasites and PfHRP2.

**FIG 2:**
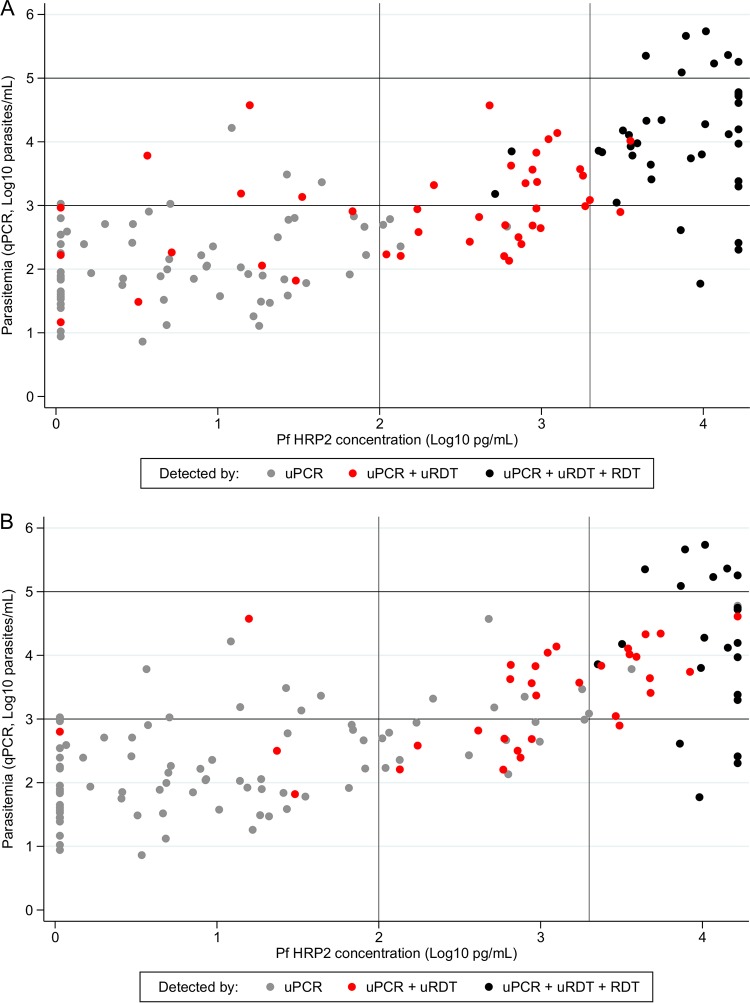
Increased range of PfHRP2 detection in uRDT compared to RDT and corresponding increase in detection of lower parasitemias for rapid tests performed in the laboratory (A) or in the field (B). Parasitemia measured by uPCR and the corresponding PfHRP2 concentration by Quansys ELISA are presented for samples positive for P. falciparum only by uPCR (monospecific infection) and by type of detection method. Vertical lines indicate PfHRP2 concentrations of 100 and 2,000 pg/ml, while horizontal lines correspond to 1,000 and 100,000 parasites/ml (see the text). Mixed P. falciparum plus P. vivax uPCR-positive samples and Plasmodium DNA-positive samples with PfHRP2 are presented in Fig. S1 in the supplemental material.

The qualitative increase in sensitivity of the uRDT compared to standard RDT and microscopy was verified in terms of quantitative PfHRP2 detection by Quansys ELISA and parasitemia by uPCR. Quantitative analysis was performed on samples with a monospecific P. falciparum infection by uPCR. PfHRP2 was also quantified in these samples by Quansys ELISA (Fig. 2). In this population of asymptomatic carriers, the P. falciparum infections detected by uPCR had low densities ranging from 10 to 544,666 parasites per ml (p/ml) (geometric mean, 734 p/ml; 95% CI, 481 to 1,117 p/ml).

In the laboratory, the geometric mean parasitemia detected by uRDT was 3,019 p/ml (95% CI, 1,790 to 5,094 p/ml; *n* = 79), compared to 11,352 (95% CI, 5,643 to 22,837 p/ml; *n* = 38) by RDT (Table 4). The uRDT performed better than the RDT to detect parasitemia between 1,000 and 10,000 p/ml (27/31 [87%] by uRDT compared to 15/31 [48%] by RDT) and between 100 and 1,000 p/ml (24/50 [48%] by uRDT compared to 3/50 [6%] by RDT). The performance of microscopy was similar to that of RDT. All three methods performed well above 100,000 p/ml (7/7 [100%]) and poorly below 100 p/ml (Table 4).

**TABLE 4:** Detection properties of the different tests by parasitemia for monospecific P. falciparum uPCR-positive infections (P. falciparum by reference B)^*a*^

Test	Parasitemia (no. of parasites/ml)^*b*^		No. of infections detected by parasitemia category (% of uPCR reference)^*c*^					
	Geometric mean (95% CI)	Median (IQR)	>100,000 p/ml	10,000–100,000 p/ml	1,000–10,000 p/ml	100–1,000 p/ml	<100 p/ml	Total
Lab uRDT	3,019 (1,790–5094)	2,939 (484–13,748)	7 (100)	17 (94)	27 (87)	24 (48)	4 (11)	79 (55)
Lab RDT	11,352 (5,643–22,837)	11,144 (4,377–52,429)	7 (100)	12 (67)	15 (48)	3 (6)	1 (3)	38 (27)
Microscopy	19,132 (10,701–34,205)	15,151 (6,919–54,225)	7 (100)	16 (89)	12 (39)	1 (2)	0 (0)	36 (25)
uPCR reference	734 (481– 1,117)	497 (90–5,506)	7 (100)	18 (100)	31 (100)	50 (100)	37 (100)	143 (100)
Field uRDT	4,960 (2,657–9,260)	6,764 (632–18,924)	7 (100)	15 (83)	17 (57)	14 (29)	2 (6)	55 (39)
Field RDT	14,237 (4,659–43,509)	15,229 (2,410–170,102)	7 (100)	7 (39)	5 (17)	3 (6)	1 (3)	23 (16)
uPCR reference	742 (485–1135)	493 (95–4,941)	7 (100)	18 (100)	30 (100)	49 (100)	36 (100)	140 (100)

a Corresponding graphs are presented in Fig. S2 in the supplemental material.

b 95% CI, 95% confidence interval; IQR, interquartile range.

c Parasitemia is given in the number of parasites per milliliter (p/ml).

The median PfHRP2 concentration detected was 1,991 pg/ml (IQR, 588 to 8,377 pg/ml; *n* = 75) by uRDT compared to 8,377 pg/ml (IQR, 3,910 to 16,500 pg/ml; *n* = 37) by RDT (Table 5). The uRDT detected 100% (37/37) of samples above 2,000 pg/ml, 88% (29/33) of samples between 100 pg/ml and 2,000 pg/ml, and 19% (9/47) of samples with detectable HRP2 below 100 pg/ml. In contrast, the RDT detected 95% (35/37), 6% (2/33), and 0% (0/47), respectively (Table 5).

**TABLE 5:** Detection properties of the different tests by concentration for monospecific P. falciparum uPCR-positive infections (P. falciparum by reference B)

Test	Median HRP2 concn (pg/ml) (IQR)	No. of infections detected by category of HRP2 concn (% of uPCR reference)			
		>2,000 pg/ml	100–2,000 pg/ml	<100 pg/ml	Total
Lab uRDT	1,991 (588–8,377)	37 (100)	29 (88)	9 (19)	75 (64)
Lab RDT	8,377 (3,910–16,500^*a*^)	35 (95)	2 (6)	0 (0)	37 (32)
Microscopy	4,780 (3,187–14,216)	27 (73)	6 (18)	2 (4)	35 (30)
uPCR reference	517 (19–3,558)	37 (100)	33 (100)	47 (100)	117 (100)
Field uRDT	3,910 (882–10,378)	34 (94)	16 (48)	3 (7)	53 (47)
Field RDT	12,937 (7,811–16,500^*a*^)	22 (61)	0 (0)	0 (0)	22 (19)
uPCR reference	553 (19–3,648)	36 (100)	33 (100)	45 (100)	114 (100)

a The upper limit of quantification of the reference test was 16,500, which was reached in a total of 17 samples.

In the field, the geometric mean parasitemia and median HRP2 concentration detected were higher: for uRDT, 4,960 p/ml and 3,910 pg/ml; for RDT, 14,237 p/ml and 2,937 pg/ml (Table 4).

**FIG 3:**
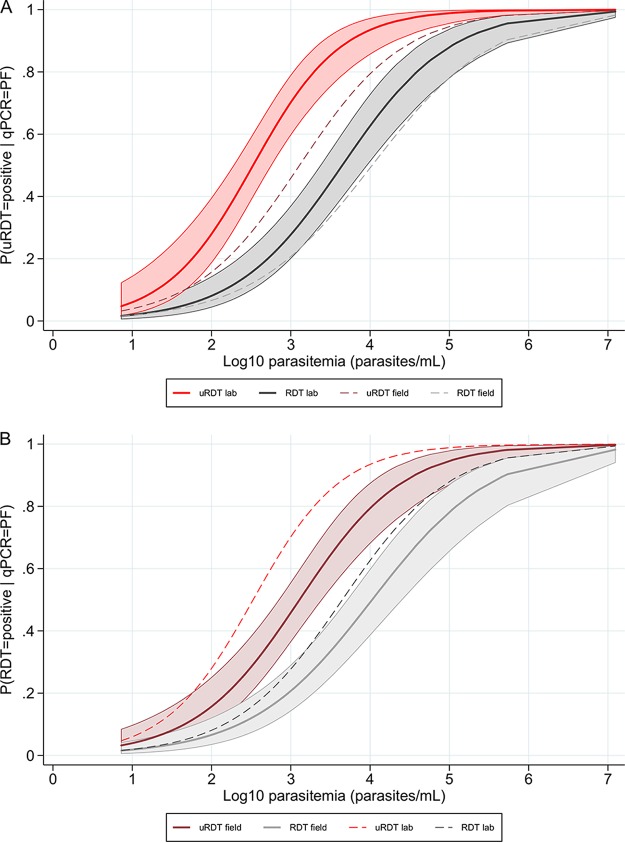
Probability of a P. falciparum-positive test for uRDT compared to RDT according to the parasitemia in uPCR-positive single P. falciparum infections. (A) Lab test results (solid line) and 95% CI (shaded area); field test results (dashed line). (B) Field test results (solid line) and 95% CI (shaded area); lab test results (dashed line).

The probability of a monospecific P. falciparum infection to be given a positive rapid test result increased with parasitemia. In the laboratory, the probability of a positive test was significantly higher for uRDT than for RDT for infections between 50 and 300,000 p/ml (Fig. 3A). In the field, the probability of a positive test was significantly higher for uRDT than for RDT for infections between 200 and 100,000 p/ml (Fig. 3B). At any parasitemia, the probability of a positive test was lower in the field than in the laboratory (Fig. 3A and B).

### Readability.

**FIG 4:**
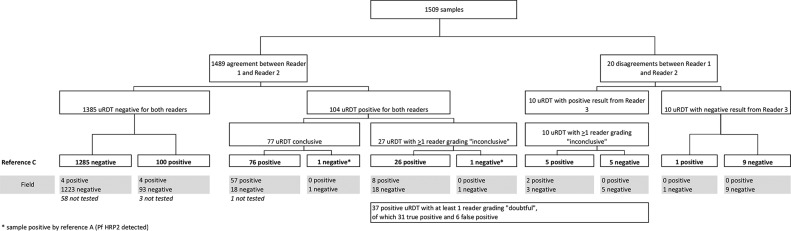
Readability of the uRDT in the laboratory. Agreement between readers, semiquantitative assessment of signal intensity, corresponding results according to reference C, and matching results of field uRDT testing.

In the laboratory, the two independent readers interpreting uRDT agreed on the results of 1,489 out of 1,509 samples (98.7%), of which 1,385 samples were interpreted negative and 104 positive. Of the 104 uRDT-positive readings, there were 102 P. falciparum infections and 2 false-positive results compared to reference C. Of the 20 discordant results between the first and second reader, 6 corresponded to P. falciparum infections, and 14 were negative. For these discordant results, the third reader interpeted 5/6 as P. falciparum infections and 5/14 as negative (Fig. 4).

The positive signals were conclusive according to both readers in 77/104 (74.0%) concordant uRDT readings, corresponding to 76 P. falciparum infections and 1 false-positive result compared to reference C. Among the remaining 37 positive results by uRDT (27 concordant and 10 discordant results interpreted positive by reader 3), 13 were graded “inconclusive” by one reader only and 24 were graded “inconclusive” by both readers.

In total, 31 (29%) of 107 true P. falciparum infections identified by uRDT in the laboratory had very low intensity signals (12 graded “inconclusive” once and 19 twice). Misinterpretation of negative tests as very low intensity positive results also resulted in 6/7 false-positive results. The field tests corresponding to samples with low intensity in the laboratory were much less likely to be interpreted as positive: only 30% (8/26) of tests where at least one reader gave an inconclusive result compared to 75% (57/76) for samples with conclusive interpretation in the laboratory (Fig. 4).

## DISCUSSION

### Summary.

In this study, >1,500 asymptomatic individuals were screened using the uRDT in a low-incidence setting of eastern Myanmar. The performance of the uRDT against combined uPCR and Quansys ELISA (reference C) showed a twofold increase in sensitivity compared to that of the RDT, 51.4% versus 25.2%, respectively. The specificities of the two rapid tests were similar, 99 to 100%. This twofold increase in sensitivity was also observed for performance against Quansys ELISA (reference A) and uPCR (reference B). It resulted from an improved detection of low-density infections between 100 and 10,000 p/ml. The sensitivity of uRDT was still higher than the sensitivity of RDT in the field, but both were lower than the same test performed in the laboratory.

### Limitations and biases.

The study was conducted as part of prevalence surveys routinely aimed at “hotspot” identification within an elimination program (22). The surveys were conducted in different seasons in 35 villages of different malaria intensity profiles, ranging from 0 to >40% P. falciparum prevalence based on uPCR detection (23). At the individual level, the venous blood surveys involved a random fraction of the adult population. In the smaller villages (e.g., 20 households, ∼100 inhabitants), the sample may have been similar to a continuous series of villagers when nearly 50% of the adult population was sampled. However, since only asymptomatic participants were included in this analysis, it is unlikely that sampling could introduce a bias related to individual response to Plasmodium infections, which could potentially affect the test result.

The evaluations reported here correspond to two very different conditions: in the laboratory, two independent expert laboratory technicians evaluated the test under optimal conditions. The field survey team members received a 2-day training on uRDT before the start of their activities. A small team of six persons shared a heavy workload to obtain participant consent, collect demographic data, test all participants using RDT and uRDT, and collect venous blood from the subsample of participants. No dedicated workspace was available when surveys were conducted door to door. The availability of adequate light sources may also have been an issue where active rural populations were often most available in the early morning or in the evening, outside their daily activities.

As a result, it is evident that additional attention is needed to identify very faint signals as positive results: approximately 30% (37/114, of which 31 were also uPCR positive) of uRDTs interpreted as positive by two laboratory readers involved at least one of them grading the signal as “inconclusive” or doubtful, and only 30% (10/31) of these were identified in field conditions. Better training materials may be required to ensure that field users of the tests can more confidently interpret weak signals.

Using capillary blood instead of venous blood might have led to a lower sensitivity of the uRDT in the field than in the laboratory. However, a similar decrease in sensitivity was measured for the RDT, with field testing also detecting close to 60% of laboratory-positive samples. No difference was previously shown between results for capillary and venous blood testing for microscopically detectable parasitemia (24). This seems to confirm that the lower sensitivity might be related, rather, to readability issues associated with faint signals in the field.

### Generalizability.

In a pilot study in the same setting, the sensitivity and specificity of the uRDT compared to uPCR were estimated to be 44% and 99.8%, respectively, based on only nine P. falciparum infections detected by uPCR, of which four were PfHRP2 positive, and zero were positive by RDT (20). The present study estimated the sensitivity and specificity more precisely and demonstrated a twofold increase in detection by uRDT compared to RDT or microscopy. The performance for both uRDT and RDT under field conditions was approximately 40% lower than in the laboratory. It will be important to understand what interventions such as proficiency testing or better job aids could improve the operational performance of RDTs in the context of surveys.

Both uRDT and RDT detected a significant number of infections below their lower limit of detection (LLOD), which leads to the geometric mean parasitemia detected being lower than the LLOD. This is potentially driven by the HRP2 antigen dynamics in the blood, which results in a nonstoichiometric relationship between HRP2 and parasitemia.

Indeed, HRP2 persists in the bloodstream following parasite clearance for at least 7 days and up to several weeks (25, 26). A high-sensitivity HRP2-based assay raised concerns that it would detect residual HRP2 in the absence of parasites and thus increase the diagnosis of resolved infections. Out of 114 samples positive by uRDT in this study, only 2 had confirmed PfHRP2 antigens by Quansys ELISA but no Plasmodium DNA by uPCR. In contrast, Quansys ELISA detected HRP2 associated with P. falciparum DNA in 96/114 samples and with P. vivax DNA or undetermined Plasmodium DNA in 7/114 samples. HRP2 persistence was therefore not a major source of misdiagnosis among asymptomatic adults in this context.

**FIG 5:**
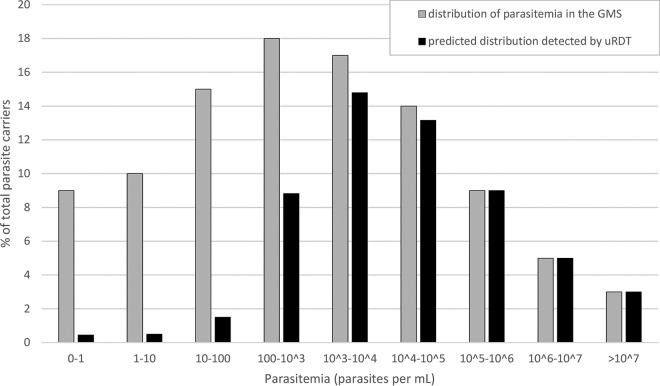
Theoretical distribution of P. falciparum parasitemia among carriers in the Greater Mekong Subregion (reference data from Imwong et al. [3] shown by the gray bars) and the corresponding fraction of each class that would be detected by uRDT (Table 4, laboratory uRDT results shown by the black bars). If used in optimal conditions, uRDT is predicted to detect up to 56% of the carriers in the GMS setting.

Given the distribution of parasitemia proposed by Imwong et al. (3) and the results obtained here, we extrapolate that the uRDT will be able to detect approximately 50% of all P. falciparum infections in GMS settings (Fig. 5). Standard RDT and microscopy already estimate reliably the number of carriers harboring parasitemias of ≥10,000 parasites/ml (Table 4). The improvement from uRDT comes from detecting infections with densities between 100 and 10,000 parasites/ml, a range of parasitemia which includes 35% of all infected carriers (Fig. 5).

### Implications.

Quality evidence regarding the size of the low-density infectious reservoir acquired directly in the field could simplify the targeting of interventions for the rapid elimination of P. falciparum malaria and bring the decision-making closer to the field level (27). However, strategies targeting asymptomatic carriers will be widely adopted by national malaria control programs only if they rely on a simple, field-robust, and inexpensive diagnostic test to measure malaria prevalence and/or identify individuals harboring low-density P. falciparum infections. The uRDT evaluated here shows promising qualities in terms of cost (approximately 1 U.S. dollar/test), of testing instructions compared to the standard RDT, and of the range of low-density infections detected.

The uRDT will improve the accuracy of reservoir size measurement over RDT differently between contexts, since the distribution of low- versus high-density infections is determined by the epidemiology of malaria. In this study, the detection of >50% of uPCR-positive samples suggests that uRDT could be an adequate survey tool to estimate accurately the size of the low-density asymptomatic reservoir in the GMS (Fig. 5).

Given its increased performance compared to RDT, uRDT is also expected to improve effectiveness of mass screening and treatment (MSAT) interventions in low-incidence settings. In eastern Myanmar, MDA with a median 90% participation resulted in a median 90% decrease of prevalence in villages also accessing malaria early diagnosis and treatment at the community level (7, 23). With a similar 90% participation in an MSAT intervention, approximately 45% of P. falciparum-infected carriers in the village would be detected, resulting at best in a 50% decrease in prevalence. However, given the range of parasitemia detected by uRDT, >95% of P. falciparum parasite biomass at the time of MSAT would be destroyed. Predicting the impact of an uRDT-based MSAT addressing only 50% of carriers is out of the scope of this study. It involves local transmission dynamics, which could be targeted by additional vector control interventions, and complex within-host interactions (duration of carriage, spontaneous clearance, parasite density oscillations), which have recently begun to be investigated using highly sensitive methods (12).

Finally, as recently confirmed, a significant limitation of the uRDT is that it cannot detect P. falciparum infections with *hrp2 hrp3* double deletion mutations (21). While *hrp2 hrp3* double deletion mutations are not frequent in the GMS and seem to be fairly localized, this limitation could be addressed by including a second equally sensitive marker of P. falciparum infection on the same test (28, 29).

### Conclusion.

This study tested the performance of a new uRDT in the laboratory and in the field using a large set of well-characterized surveys conducted within a P. falciparum elimination program in the GMS. The uRDT detected close to 50% of estimated P. falciparum infections, a twofold improvement compared to the numbers previously detected by standard RDT or microscopy. Field and *in silico* evaluations of uRDT in specific use cases, such as prevalence estimation, reactive case detection, or MSAT, and in different settings, are now necessary to define its future role in elimination strategies.

## Supplementary Material

Supplemental material
