# Supplementary material for: Operational Performance of a Plasmodium falciparum Ultrasensitive Rapid Diagnostic Test for Detection of Asymptomatic Infections in Eastern Myanmar
Source: J Clin Microbiol. 2018 Jul 26;56(8):e00565-18. doi: 10.1128/JCM.00565-18 (PMC6062819; doi:10.1128/JCM.00565-18)
Supplement: Supplemental material [file supp_56_8_e00565-18__index.html]

Supplemental material 

# Operational Performance of a Plasmodium falciparum Ultrasensitive Rapid Diagnostic Test for Detection of Asymptomatic Infections in Eastern Myanmar

## Supplemental material

- Supplemental file 1 -

  Fig. S1 (Increased range of PfHRP2 detection by uRDT compared to results for RDT and corresponding increase in detection of lower parasitemias) and S2 (Number of monospecific *P. falciparum* infections detected by uPCR and by each method according to parasitemia categories defined by uPCR) and Table S1 (Color-coded version of Table 3)

  PDF, 421K
