# Supplementary material for: Operational Performance of a Plasmodium falciparum Ultrasensitive Rapid Diagnostic Test for Detection of Asymptomatic Infections in Eastern Myanmar
Source: J Clin Microbiol. 2018 Jul 26;56(8):e00565-18. doi: 10.1128/JCM.00565-18 (PMC6062819; doi:10.1128/JCM.00565-18)
Supplement: Supplemental material [file JCM.00565-18_zjm999096058s1.pdf]

Supplementary figures

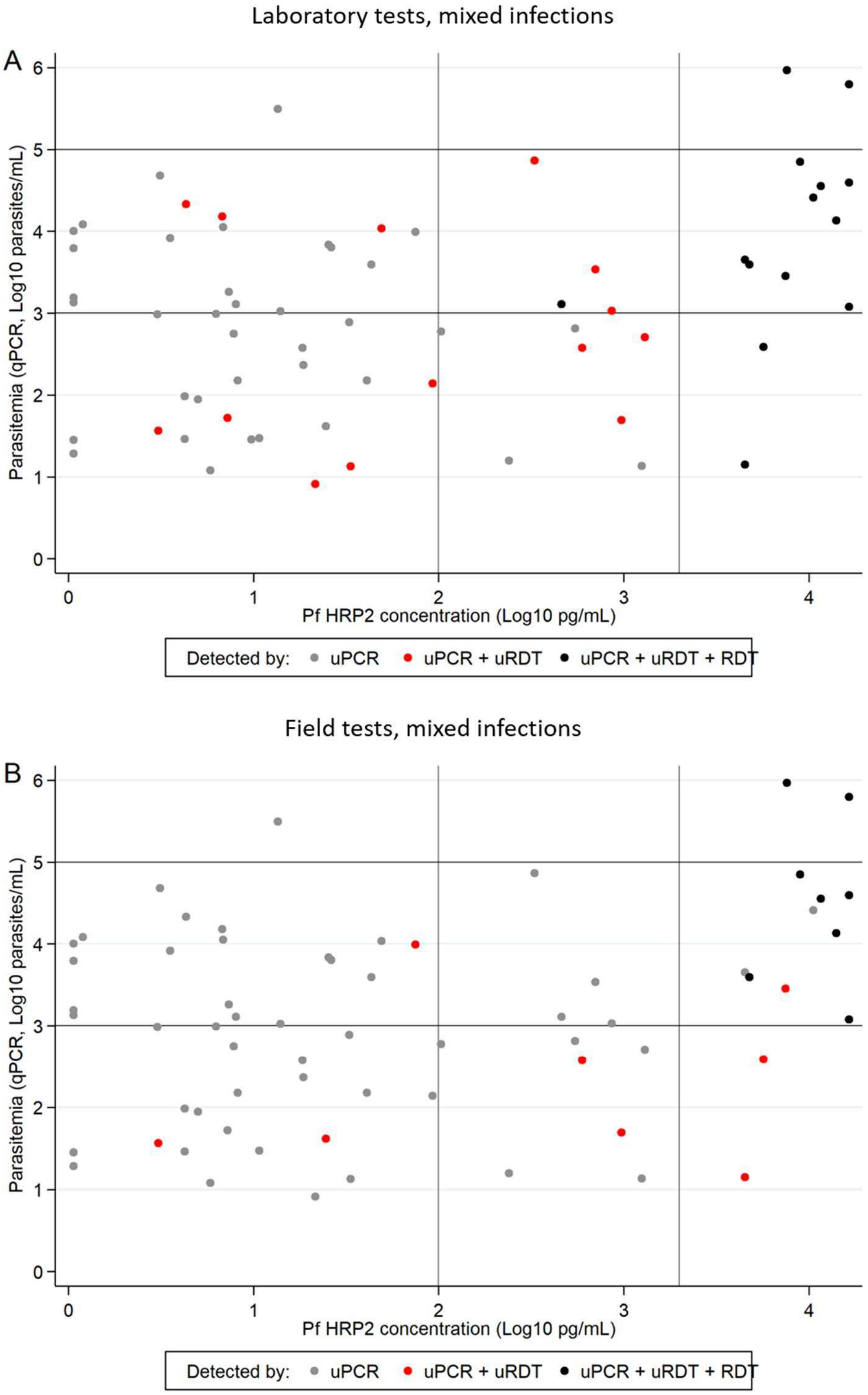

Supplementary figure 1: Increased range of PfHRP2 detection in uRDT compared to RDT and corresponding increase in detection of lower parasitemias, for rapid tests performed A: in the laboratory or B: in the field.

Parasitemia measured by uPCR and corresponding PfHRP2 concentration by Quansys ELISA are presented for samples identified as mixed *P. falciparum* + *P. vivax* by uPCR or as Plasmodium DNA positive samples with PfHRP2. Vertical lines indicate PfHRP2 concentrations of 100 and 2000 pg/mL, while horizontal lines correspond to 1,000 and 100,000 parasites/mL (see text).

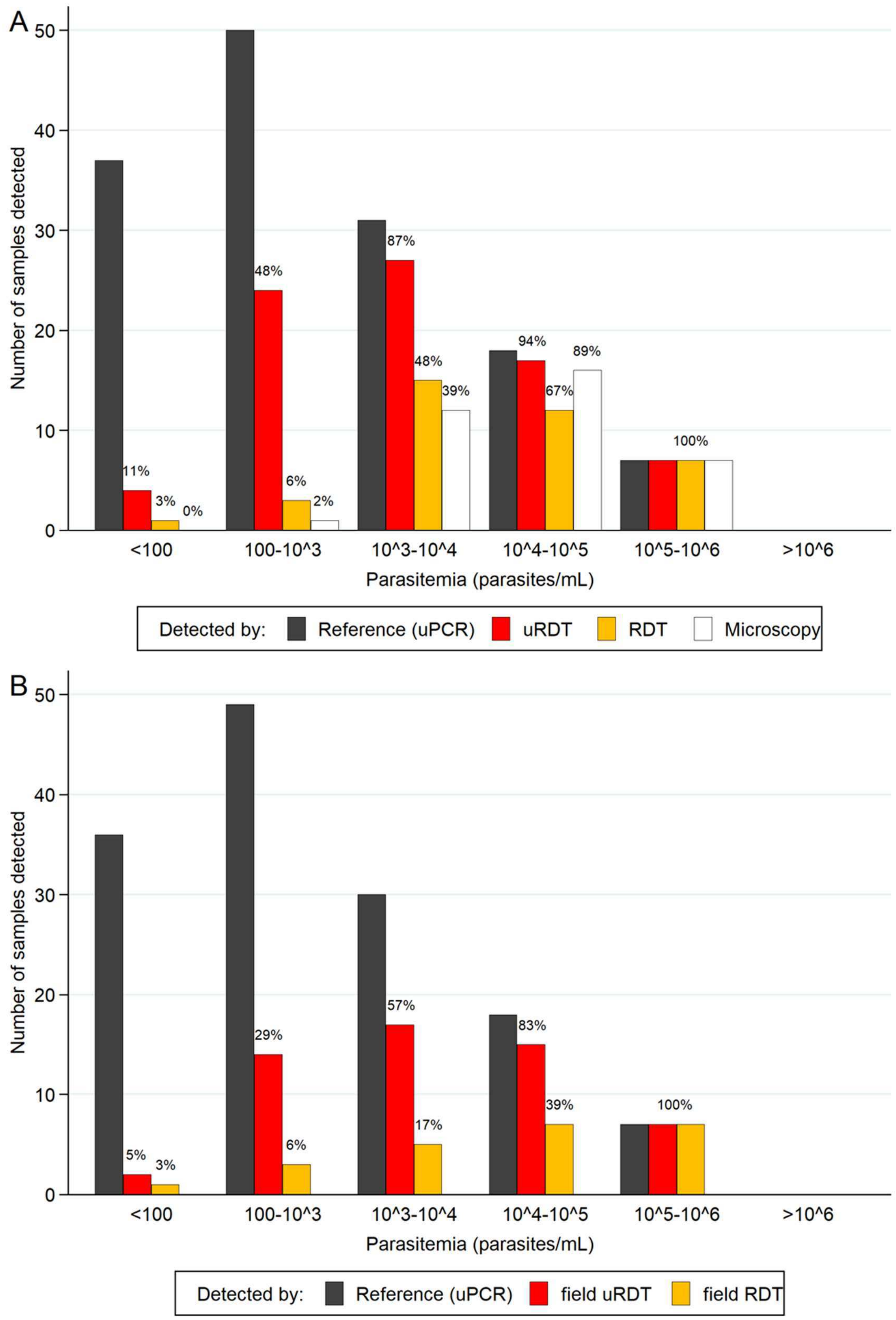

Supplementary figure 2: Number of monospecific *P. falciparum* infections detected by uPCR (reference) and by each method according to parasitemia categories defined by uPCR (graphical representation of Table 4). A: comparison of uRDT, RDT and microscopy tests conducted in the laboratory. B: comparison of uRDT and RDT tests conducted in the field. The corresponding percentage of uPCR positive detected by each method is indicated above each bar.

Supplementary Table 1: Colour-coded version of Table 3 presenting the number of samples per reference result for all samples with a *P. falciparum* positive uRDT and a *P. falciparum* negative result for at least 1 reference (A-C).

Red(1): *P. falciparum* negative samples by all 3 reference methods. These are most likely false positive uRDT.

Orange(2): *Pf*HRP2-positive, *Pf*DNA negative, and *Plasmodium* DNA negative samples (positive by reference A only). These 2 samples could correspond to recently cleared infections with persisting HRP2 antigenemia.

Yellow(3): *Pf*HRP2 positive, *Pf* DNA negative, and *Plasmodium* DNA positive samples (*P. falciparum* positive by reference A and C)

Green(4): *Pf*HRP2 negative and *Pf* DNA positive (*P. falciparum* positive by reference B and C).

n.a: not applicable, corresponds to *Pf*HRP2 positive and *Pf* DNA positive samples (true positive according to all references).

Red+Orange = false-positive by reference C (n=7 for laboratory and n=4 for field);
Green+Red = false-positive by reference A (n=9 for laboratory and n=6 for field);
Yellow+Orange+Red = false-positive by reference B (n=14 for laboratory and n=8 for field).

LABORATORY				
	uPCR result (type of DNA identified)			
Quansys ELISA result (type of antigens identified)	None	*P. vivax*	*Plasmodium* spp.	*P. falciparum*
None	3(1)	1(1)	0(1)	2(4)
P. vivax (PvLDH)	0(1)	1(1)	0(1)	0(4)
P. falciparum (PfHRP2)	2(2)	0(3)	4(3)	n.a
P. falciparum and *P. vivax* (PfHRP2+PvLDH)	0(2)	1(3)	2(3)	n.a
Plasmodium. spp (Pan LDH only)	0(1)	0(1)	0(1)	2(4)
**FIELD**				
	uPCR result (type of DNA identified)			
Quansys ELISA result (type of antigens identified)	None	*P. vivax*	*Plasmodium* spp.	*P. falciparum*
None	1(1)	1(1)	0(1)	0(4)
P. vivax (PvLDH)	0(1)	2(1)	0(1)	1(4)
P. falciparum (PfHRP2)	0(2)	1(3)	3(3)	n.a
P. falciparum and *P. vivax* (PfHRP2+PvLDH)	0(2)	0(3)	0(3)	n.a
Plasmodium. spp (Pan LDH only)	0(1)	0(1)	0(1)	1(4)
